# The Structure and Mechanical Properties of High-Strength Bulk Ultrafine-Grained Cobalt Prepared Using High-Energy Ball Milling in Combination with Spark Plasma Sintering

**DOI:** 10.3390/ma9050391

**Published:** 2016-05-19

**Authors:** Ivo Marek, Dalibor Vojtěch, Alena Michalcová, Tomáš František Kubatík

**Affiliations:** 1 Department of Metals and Corrosion Engineering, University of Chemistry and Technology Prague, Technická 5, 166 28 Prague 6, Czech Republic; dalibor.vojtech@vscht.cz (D.V.); alena.michalcova@vscht.cz (A.M.); 2 Institute of Plasma Physics AS CR, v.v.i., Za Slovankou 1782/3, 182 00 Prague 8, Czech Republic; kubatik@ipp.cas.cz

**Keywords:** ultrafine-grained material, cobalt, ball milling, spark plasma sintering, mechanical properties

## Abstract

In this study, bulk ultrafine-grained and micro-crystalline cobalt was prepared using a combination of high-energy ball milling and subsequent spark plasma sintering. The average grain sizes of the ultrafine-grained and micro-crystalline materials were 200 nm and 1 μm, respectively. Mechanical properties such as the compressive yield strength, the ultimate compressive strength, the maximum compressive deformation and the Vickers hardness were studied and compared with those of a coarse-grained as-cast cobalt reference sample. The bulk ultrafine-grained sample showed an ultra-high compressive yield strength that was greater than 1 GPa, which is discussed with respect to the preparation technique and a structural investigation.

## 1. Introduction

Research on nano-crystalline (with grain sizes between 10 and 100 nm) and ultrafine-grained (with grain sizes up to 500 nm) polycrystalline metals and alloys has evolved considerably over the last few decades [1,2,3]. These materials exhibit unique properties that are lacking in their coarse-grained counterparts. Such properties predispose them to use in a wide range of magnetic, optical and structural applications. It is well known that these properties are related to morphology, crystallite size and inter-crystallite interactions [4,5,6]. There are two basic methods of producing these materials; one is top-down, and the other is bottom-up. The top-down approach includes the structural disintegration of bulk materials by means of severe plastic deformation (SPD) techniques such as high-pressure torsion (HPT) [7], equal channel angular pressing (ECAP) [8], surface mechanical attrition treatment (SMAT) [9] and high-energy ball milling (BM). In contrast, the bottom-up method involves either one-step processes such as electrodeposition or two-step preparation, which consists of nanoparticle synthesis followed by consolidation [10]. In this study, we used a combination of these two approaches. High-energy ball milling was used to prepare the nano-crystalline powder; it was followed by consolidation using spark plasma sintering.

During high-energy ball milling, particles are subjected to repeated flattening, cold welding, fracturing and rewelding. When two milling balls collide, a certain amount of powder is trapped between them, and the force of the impact plastically deforms the powder, which leads to strain hardening and fracture. The processes that lead to formation of nano-crystalline structures during milling have been described in a few reports [11,12,13]. It has been reported that in the early stages of milling, shear bands result from the high deformation rates. These shear bands contain a large number of dislocations and have approximate widths of 0.5–1.0 μm. Further milling increases the dislocation density, which increases the average atomic strain. At a certain dislocation density, a crystal disintegrates into subgrains that are separated by low-angle grain boundaries, and the lattice strain decreases. As processing continues, the grain size tends to decrease steadily, and the shear bands merge. The low-angle grain boundaries are replaced by high-angle boundaries and, grain rotation occurs. This has been shown by the absence of texture in electron diffraction patterns and random orientations of the grains. As a result, dislocation-free nano-crystalline grains are formed [14,15]. Other studies model the minimum grain size achievable by ball milling. Some of them say that reducibility is the result of competition between the plastic deformation that results from the motion of dislocations and the recovery and recrystallization behaviour of the material [16,17,18]. It has also been reported that the minimum grain size is inversely related to the melting point and stacking fault energy of fcc Cu, Ni, Ag and Al [17]. However, the minimum grain sizes of other fcc metals and all bcc and hcp metals showed almost no dependence on the melting point [19,20]. Nevertheless, further investigations are needed before serious explanations can be made.

In the field of powder metallurgy, many methods of compacting have been proposed. As an example, hot pressing, hot isostatic pressing or hot extrusion can be mentioned [21,22,23]. However, these techniques require relatively high temperatures and long sintering times, which significantly limit their usability for processing nano-crystalline bulk materials. Spark plasma sintering (SPS) is a novel technique that allows bulk materials to be fabricated from nano-crystalline powders. SPS is a pressure-driven consolidation method that operates with a pulsed direct current that passes through the powder sample, which is placed in a graphite die. The current pulses cause very quick heating of the sample (up to 1000 °C/min) and, in combination with the applied pressure, cause fast consolidation of the powder, which effectively hinders the undesirable coarsening of the grains. One of the other benefits of this method is the possibility of producing bulk materials with densities close to their theoretical values. Such materials exhibit significantly improved mechanical properties [24,25,26,27,28,29,30,31,32,33]. For example, studies focusing on Al-based alloys prepared by this technique reporting a compressive strength of 1200 MPa have been published [25,29].

## 2. Results

**Figure 1:**
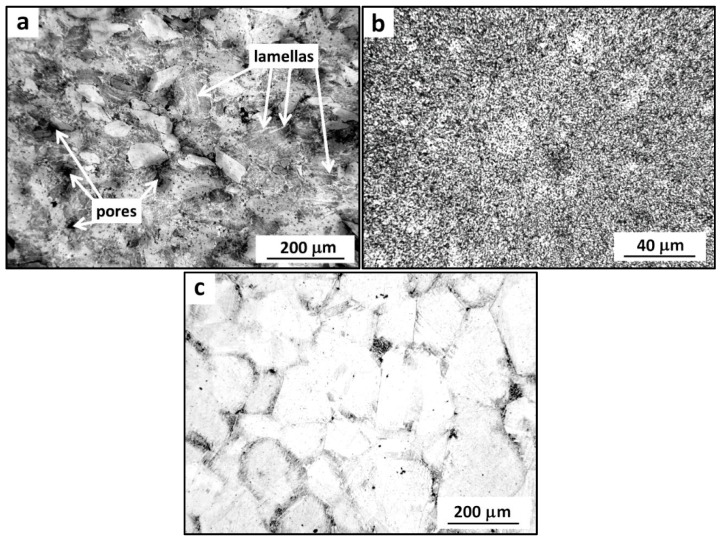
Microstructures of the bulk cobalt samples (OM): (**a**) ultrafine-grained cobalt prepared by ball milling and spark plasma sintering; (**b**) micro-crystalline cobalt prepared by spark plasma sintering; and (**c**) coarse-grained as-cast cobalt.

In this study, bulk cobalt prepared using various techniques, including high-energy ball milling and spark plasma sintering, was investigated. In Figure 1, optical microscopy micrographs show the microstructures of the prepared samples. In Figure 1a, the structure of the cobalt sample prepared using a combination of ball milling and subsequent spark plasma sintering is visible. It consists of particles with an average size of approximately 100 μm and residual pores, which are shown as black spots and dots. The average porosity of this sample was obtained using image analysis and reached approximately 9.6%. Alternating brighter and darker stripes inside the particles denote its internal lamellar structure (shown in Figure 1a) which is a typical result of ball milling. These particles were expected to have ultrafine-grained internal structure on the scale of nanometres and therefore, further investigation was performed by means of transmission electron microscopy. The microstructure of the micro-crystalline bulk sample is shown in Figure 1b. It contains a visible homogenous structure consisting of bonded powder particles. In this case, no internal structure of the particles was observed, which leads to the assumption that the grain size corresponds to the particle size (approx. 1 μm). The average porosity of this sample reached 0.3%. Figure 1c shows the microstructure of the coarse-grained as-cast cobalt sample. It was found that the average grain size of this material reached 190 μm and the average porosity was 1.2%. Relative densities of the samples were determined from their dimensions and weight and summarized in Table 1. All calculated density values were in a good agreement with the average porosity of the samples obtained by image analysis.

**Figure 2:**
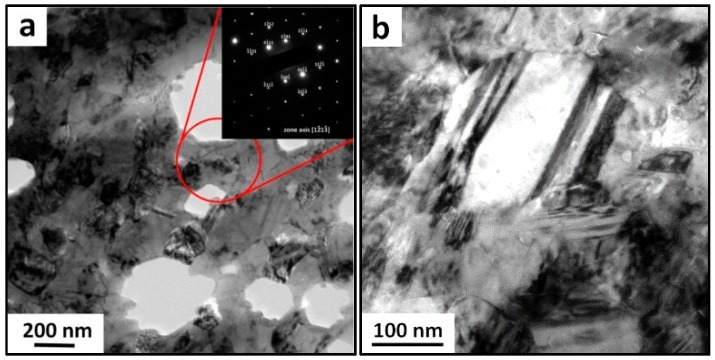
TEM micrographs of bulk ultrafine-grained cobalt. (**a**) an overview micrograph with SAED pattern taken from marked area; (**b**) a detailed view of single grain.

A TEM micrograph of the ultrafine-grained cobalt sample and a selected area diffraction pattern (SAED) are shown in Figure 2a. It can be observed that the microstructure is composed of equi-axed grains that have an average size of approximately 200 nm. The holes in the sample come from preparation by the Gatan precision ion polishing system (PIPS) and they cannot be considered as pores. The SAED pattern taken from marked area comprises diffraction maxima indicating the hexagonal structure in this area. The detailed view of the microstructure shown in Figure 2b reveals also the presence of deformation twins in appropriately oriented grains that originate from ball milling (dark stripes across the grain).

**Figure 3:**
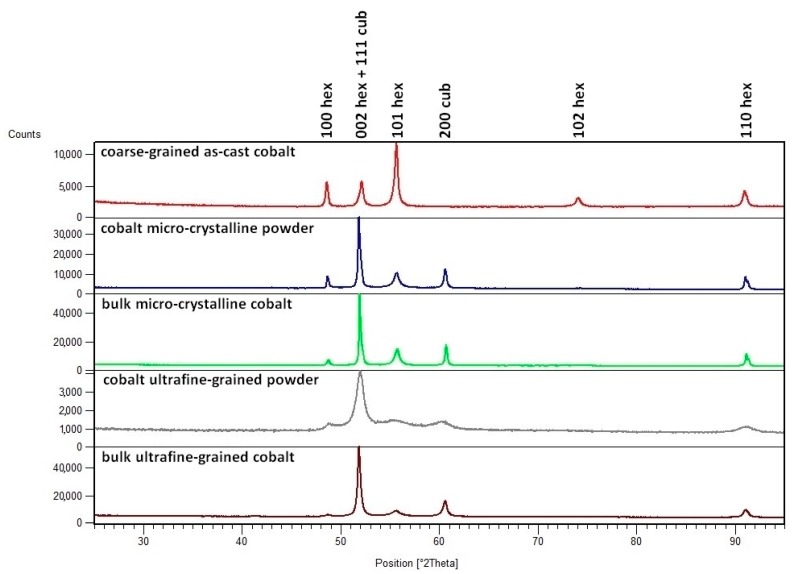
XRD patterns of the powders and the bulk materials.

Figure 3 represents XRD diffraction patterns of bulk materials and powder precursors. The reference as-cast cobalt sample only contained hcp structures, while the initial powders and the prepared compacts contained a mixture of hcp and metastable fcc structure. The percentage amount of fcc and hcp phases was determined by X’Pert HighScore Plus program (PANalytical B.V., Almelo, The Netherlands). The raw commercial micro-crystalline powder contained 70% of fcc phase and 30% of hcp phase. Ball-milled powder was 60% composed of the hcp and 40% of the fcc structure. Bulk micro-crystalline and bulk ultrafine-grained samples contained 61% of fcc and 39% of hcp, and 59% of fcc and 41% of hcp structure, respectively. Comparing the diffraction patterns of powders clearly shows that pattern of conventional micro-crystalline cobalt contains relatively sharp peaks, whereas the pattern of the ball-milled powder contains very broad peaks. The observed peak broadening can be attributed to significant grain refinement, internal stress and lattice defects in the cobalt powder that were induced by the ball-milling process. In contrast, the diffraction pattern of the bulk ultrafine-grained cobalt exhibits peaks that are narrower than those of ultrafine-grained powder. An explanation can be found in the partial recrystallization and grain coarsening that occurs during SPS, even though these processes are strongly limited by the short sintering time. Average crystallite size was determined using Scherrer’s calculator and reached approximately 40 ± 9 nm for hcp and 108 ± 17 nm for fcc. These results are in good agreement with lamella size observed in grains by TEM.

**Figure 4:**
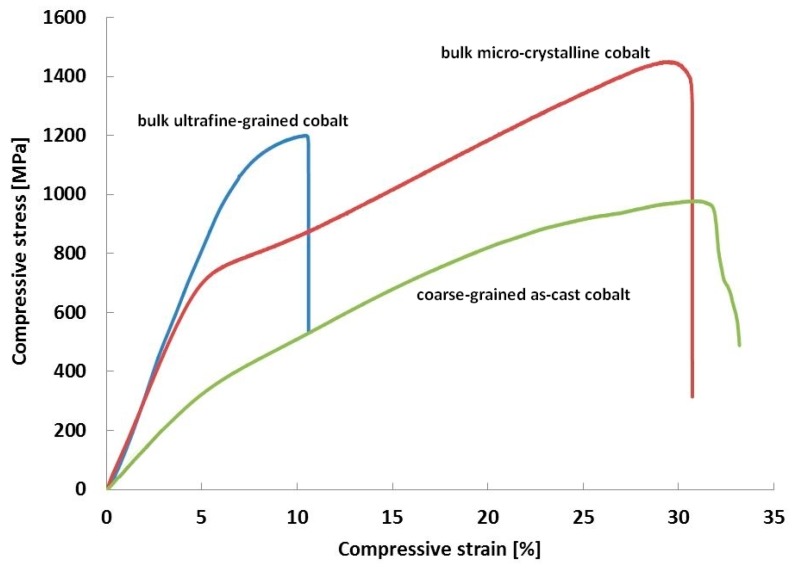
Compressive stress-strain curves for the bulk ultrafine-grained, micro-crystalline and coarse-grained as-cast cobalt sample.

The compressive stress-strain curves of the bulk materials are shown in Figure 4, and the mechanical properties are summarized in Table 1. The ultrafine-grained cobalt prepared by ball milling and SPS reached a very high compressive yield strength (CYS) of 1030 MPa. This value is more than three times that of conventional coarse-grained as-cast cobalt (340 MPa) and almost 400 MPa higher than that of the micro-crystalline cobalt consolidated by SPS (640 MPa). Similar trends are observed for the hardness. The hardness of the ultrafine-grained, micro-crystalline and as-cast samples reached 310 HV, 190 HV and 100 HV, respectively. Figure 4 and Table 1 also show that the ultimate compressive strength (UCS) of the ultrafine-grained cobalt sample was only approximately 170 MPa higher than that of CYS, and the maximum compressive deformation reached approximately 10%. In the case of the micro-crystalline cobalt, in contrast, the UCS is very high (1450 MPa), which is more than twice the CYS. A similar situation was observed for the coarse-grained as-cast sample, which has a UCS that is almost three times as large as CYS. The micro-crystalline and the as-cast samples showed maximum compressive deformations of approximately 30%. The shapes of the compressive curves for the micro-crystalline and coarse-grained as-cast cobalt samples differ from that of the ultrafine-grained cobalt sample (see Figure 4). The first two exhibit typical strain hardening across the entire region of plastic deformation. In contrast, the ultrafine-grained cobalt sample has very short region of strain hardening that is followed by macroscopic rupture.

## 3. Discussion

In this study, it is shown that high-energy ball milling combined with subsequent spark plasma sintering seems to be a suitable method of producing bulk materials with ultrafine structures and high yield strengths. Pure cobalt powder refined in a high-energy ball mill during the first step of the fabrication process was then compacted at 700 °C for 10 min with a heating rate of 100 °C/min. This operation resulted in slight grain growth, as shown by the XRD diffraction pattern (as sharpening of the peaks), because nano-crystalline and ultrafine-grained materials are generally susceptible to grain coarsening at elevated temperatures due to the high energy of the grain boundaries. However, grain coarsening is significantly reduced considering the short sintering time and high efficiency of SPS [28,34,35] (compared with other consolidation methods). The electric current running through the powder causes a rapid temperature increase at the contact points, which can even lead to partial melting. Nevertheless, in this study, areas with the coarsened structures that indicate previous melting were not observed. Ye *et al.* also reported that the flow of an electric current through the powder ameliorates and accelerates the diffusion bonding of powder particles [28].

The XRD phase analysis showed that the materials studied, except for the as-cast cobalt reference sample, consisted of mixture of hcp and fcc phases. Hcp and fcc structures can be formed from disordered polytypic structures by divergent arrangement mechanisms, and the variation between these two types of structure includes the transition from one close-packed structure to another [36,37]. The fcc-to-hcp allotropic transformation in cobalt has been identified as martensitic [38,39,40] even though its enthalpy and chemical driving force are low (−489 J/mol and −16 J/mol, respectively) [41,42], and the transformation is very gradual. That is why a certain amount of the fcc phase can be found in the cobalt even after cooling from the sintering temperature (700 °C) to room temperature [43]. The presence of fcc modifications in the initial (commercial and ball-milled) powders can be explained on the basis of interface energy, which is responsible for the stabilization of the fcc structure. It is known that decreasing the grain/particle size results in an increase of the volume of interfaces in the material. The consequence is excess energy (∆G_Int_) because of the Gibbs-Thomson effect [44],

(1) $$\Delta G_{\text{Int}}=2{\gamma V}_{m}/R$$

where γ is the interface energy; V_m_ is the molar volume; and R is the grain radius (provided that the grains are spherical). Conventional coarse-grained materials have little excess energy, and its effect on the transformation is negligible. Therefore, excess energy considerably influences the stability of fine-grained materials [45]. If we consider the excess energy, the critical activation energy (∆G) for martensitic transformation can be expressed as [42,46]:

(2) $$\Delta G=\frac{K}{{\left(\Delta g+\Delta G_{\text{Int}}\right)}^{4}}$$

where K is a constant connected with the elastic and interfacial energy between the martensitic and initial phases, and ∆g is the chemical driving force for martensitic transformation. Assuming that ∆g is negative and ∆G_Int_ is positive, the total critical activation energy increases with increasing ∆G_Int_ (the grain size is reduced). Due to the small driving energy required for fcc-to-hcp transformation, the transformation is sluggish, as was stated above, and even a slight increase of excess energy can considerably affect the transformation behavior. It has been reported that for cobalt with an average grain size of 10 μm the critical activation energy for martensitic transformation is 5 times higher than it is for conventional coarse-grained cobalt [47].

The mechanical properties were investigated using compressive tests. The bulk ultrafine-grained sample showed a compressive yield strength of 1030 MPa and a plasticity of 10%, which is relatively good, considering the generally low ductility or even brittleness of ultrafine-grained and nano-crystalline materials. Values of compressive yield strength of ultrafine-grained or nano-crystalline cobalt reported in other studies [10,36,48] are higher, even more than 1500 MPa [49]. However, plasticity of such materials reached only up to 6%. Obtained average values of the grain size were substituted into the well-known Hall-Petch relationship

(3) $$\text{CYS}=\sigma_{0}+k_{y}\times d^{-1/2}$$

where σ_0_ is the friction stress; and k_y_ is a constant referred to as the Hall-Petch slope. Multiple values of these constants for pure cobalt were found in literature, however, they differ significantly (σ_0_ = 82 MPa, k_y_ = 0.29 MN/m^3/2^ [50]; σ_0_ = 432 MPa, k_y_ = 0.19 MN/m^3/2^ [51]). The calculated values of the CYS of the ultrafine-grained sample, using the above relation and constants, are 730 and 860 MPa; they do not match the value obtained from compressive test. The same situation was observed for microcrystalline and as-cast reference samples. These differences may result from various fabrication techniques used for bulk sample preparation and grain size ranges.

It is obvious from the stress-strain curves that both the micro-crystalline sample and the coarse-grained as-cast sample show significant strain hardening. Because of presence of the fcc phase in the micro-crystalline sample, the strain hardening is expected to be the result of interactions of dislocations with grain boundaries, interaction between moving dislocations and the formation of new dislocations. Strain hardening of this sample can also be a result of increasing amount of the hcp phase in the structure as reported in [52]. It is known that cobalt with an hcp structure (in our study, the coarse-grained as-cast sample) deforms by twinning because it lacks the slip systems for dislocation movement [51,53]. The deformation twins contain relatively large numbers of non-equilibrium basal stacking faults, and the dislocations must cross-slip to other slip planes to bypass these fault-filled regions, which leads to strain hardening [54]. This mechanism probably also occurs in the hcp phase of the micro-crystalline sample. Results obtained from synchrotron X-ray diffraction reported by Wang *et al.* [55] indicate that also interfacial defects can support strain hardening. Not only the fine structure but also presence of residual pores in the ultrafine-grained sample (Figure 3a) can cause the sample to elongate less than its micro-crystalline and as-cast counterparts. The origin of the pores can be attributed to less consolidation during SPS (despite its high sintering efficiency) due to the significant work hardening experienced by the powder during ball milling, which can also lead to weaker particle-to-particle bonding. The slope of the stress-strain curve of the as-cast sample differs from those of micro-crystalline and ultrafine-grained samples. This can be ascribed to the absence of the fcc phase and lower amount of defects in the as-cast material compared with SPS processed samples. On the basis of results of the XRD phase analysis that reveal the presence of both crystallographic modifications in the ultrafine-grained specimen, it is assumed that deformation occurs by means of a combination of dislocation slipping in the fcc phase and twinning in the hcp phase, as it does in the micro-crystalline sample.

## 4. Materials and Methods

**Figure 5:**
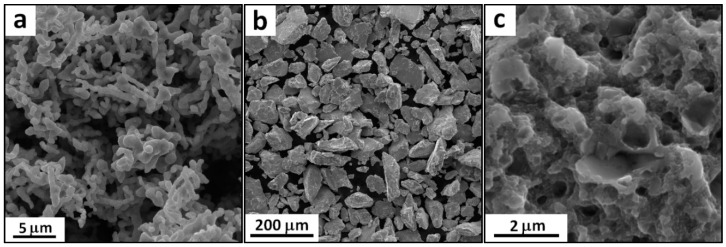
(**a**) Morphology of the conventional micro-crystalline powder; (**b**) morphology of the ball-milled powder; and (**c**) internal structure of the ball-milled powder.

In this study, two bulk cobalt samples were prepared by means of powder metallurgy (PM). As an initial material, commercially available pure micro-crystalline cobalt powder prepared by carbonyl decomposition (MERCK, Darmstadt, Germany, 99.9%, average particle size 1 μm, Figure 5a) was used. The preparation process of the first set of ultrafine-grained samples consisted of high-energy ball milling in a planetary ball mill (Retsch PM 100, Haan, Germany) followed by consolidation using the perspective method of spark plasma sintering (Thermal Technology SPS 10-4, Santa Rosa, CA, USA). High-energy ball milling was performed in a milling vessel that contained milling balls (all made of AISI 420 stainless steel). The metallic powder was milled for 2 h at a rotation speed of 400 RPM in an argon protective atmosphere. The direction of rotation was changed every 30 min. The morphology and internal structure of the ball-milled powder are shown in Figure 5b,c, respectively. The spark plasma sintering process was performed in a high-strength graphite die with an inner diameter of 20 mm at 700 °C at a heating rate of 100 °C/min. This temperature was maintained for 10 min. The pressure between the graphite rams was 80 MPa. A second set of micro-crystalline samples was prepared by SPS under the same conditions as the first set, but the initial powder was not subjected to ball milling. Coarse-grained as-cast cobalt prepared by conventional melting in an induction furnace under an argon protective atmosphere was used as a reference material. The experimental materials were investigated by means of optical microscopy (OM, Olympus PME-3, Shinjuku, Tokyo, Japan), scanning electron microscopy (SEM, TESCAN Vega, Brno, Czech Republic, LMU, W cathode, equipped with an energy dispersive spectrometer (EDS) Oxford Instruments, Abingdon, UK, Inca 350), transmission electron microscopy (TEM, Jeol JEM 3010, Akishima, Tokyo, Japan, LaB_6_ cathode, EDS Oxford Instruments, Inca 350) and XRD phase analysis (PANalytical X’Pert Pro, Almelo, The Netherlands, Co anode, HighScore Plus). The mechanical properties of the bulk samples were studied by means of compressive tests. For this purpose, cubic specimens with an edge length of 4 mm were prepared and pressed at a strain rate of 0.24 mm/min at room temperature using a universal testing machine (LabTest 5.250SP1-VM, LaborTech, Opava, Czech Republic). Vickers hardness measurements were made for a load of 30 kg.

## 5. Conclusions

In summary, bulk ultrafine-grained cobalt was successfully prepared using a combination of high-energy ball milling and subsequent spark plasma sintering, which ensured satisfactory diffusion bonding of powder particles. The prepared bulk sample exhibited compressive yield strength of 1030 MPa, which is more than three times higher that of conventional coarse-grained as-cast cobalt, but the plasticity reached only 10%. The bulk ultrafine-grained sample did not show work hardening during plastic deformation, which is the general drawback of ultrafine-grained and nano-crystalline materials.

## Figures and Tables

**Table 1:** Vickers hardness (HV), compressive yield strength (CYS), ultimate compressive strength (UCS), maximum deformation and relative density (ρ_rel_) of bulk samples.

Sample	HV	CYS (MPa)	UCS (MPa)	Max Deformation (%)	ρ_rel_ (%)
Ultrafine-grained	310 ± 4	1030	1200	10	88.8 ± 0.6
Micro-crystalline	190 ± 5	640	1450	30	98.9 ± 0.4
As-cast	100 ± 3	340	980	31	97.8 ± 0.4

## Abbreviations

The following abbreviations are used in this manuscript:

SPD	Severe plastic deformation
HPT	High pressure torsion
ECAP	Equal channel angular pressing
SMAT	Surface mechanical attrition treatment
BM	Ball milling
SPS	Spark plasma sintering
SAED	Selected area electron diffraction
PIPS	Precision ion polishing system
XRD	X-ray diffraction
HV	Vickers hardness
CYS	Compressive yield strength
UCS	Ultimate compressive strength
PM	Powder metallurgy
RPM	Revolutions per minute
OM	Optical microscopy
SEM	Scanning electron microscopy
EDS	Energy dispersive spectroscopy
TEM	Transmission electron microscopy
